# Association of timed up and go test outcomes with future incidence of cardiovascular disease and mortality in adults aged 66 years: Korean national representative longitudinal study over 5.7 years

**DOI:** 10.1186/s12877-020-01509-8

**Published:** 2020-03-19

**Authors:** Ki Young Son, Dong Wook Shin, Ji Eun Lee, Sang Hyuck Kim, Jae Moon Yun, Belong Cho

**Affiliations:** 1 Department of Family Medicine, Asan Medical Center, 88 Olympic-ro 43 gil, Songpa-gu, Seoul, 05505 Korea; 2 Department of Family Medicine & Supportive Care Center, Samsung Medical Center, Sungkyunkwan University School of Medicine, Seoul, Korea; 3 Center for Clinical Epidemiology, SAIHST, Sungkyunkwan University, Seoul, Korea; 4 Department of Family Medicine, CHA Bungdang Medical Center, CHA University, Seongnam-si, Gyeonggi-do Korea; 5 Department of Family Medicine, Bumin Hospital, Seoul, Korea; 6 Health Promotion Center, Seoul National University Hospital, Seoul, Korea; 7 Department of Family Medicine, Seoul National University Hospital, Seoul, Korea; 8 Institute on Aging, Seoul National University College of Medicine, Seoul, Korea

**Keywords:** Timed up and go test, Cardiovascular disease, Mortality

## Abstract

**Background:**

The timed up and go test (TUG) is one of the most widely used tests of mobility. We aimed to examine whether the TUG is associated with cardiovascular (CV) events, CV mortality, and all-cause mortality.

**Methods:**

Subjects in the senior cohort database of the Korean National Health Insurance Service (2002–2013) who completed the TUG as part of the National Screening Program for Transitional Ages (NSPTA) during 2007–2008 were identified. An abnormal TUG result was defined as a time ≥ 10 s. Cox proportional hazard models were used to assess the associations between TUG results and CV events, CV mortality, and all-cause mortality.

**Results:**

The mean follow-up period was 5.7 years. Incidence rates of CV events in the normal and abnormal TUG groups were 7.93 and 8.98 per 1000 person-years, while CV mortality rates were 0.96 and 1.51 per 1000 person-years, respectively. In a fully adjusted model, we found that abnormal TUG results were not associated with the incidences of CV events and CV mortality. However, abnormal TUG results (≥10 s) resulted in a 2.9-fold increase in CV mortality in women (adjusted hazard ratio 2.90, 95% confidence interval 1.15–7.30). Further, participants lacking certain CV risk factors, such as current cigarette smoking, obesity, or diabetes, had a higher CV mortality rate when TUG results were abnormal.

**Conclusions:**

Abnormal TUG results in subjects aged 66 years were associated with future CV mortality in women and in subjects without obesity, diabetes, or cigarette smoking. In patient with mobility impairment, physicians should consider CV disease risk, especially in women.

## Background

The timed up and go (TUG) test, which includes standing and walking activities common in daily life, is one of the most widely used tests of subject mobility – i.e. walking, turning and transitions. These tests are easy to perform and can assess mobility, including static balance, dynamic balance, strength in the lower extremities, and gait speed. The results of TUG tests have been shown to predict falls, fractures, hospital admission due to fractures [1], disability [2], low quality of life [3], low social participation [3], complications after elective surgery in cancer patients [4], and onset of difficulty of activity of daily living [5].

Functional decline in mobility of older adults has been found to be associated with future cardiovascular (CV) and all-cause mortality. An inability to walk 400 m [6] or slow walking speed [7, 8] is associated with a higher risk of CV disease and mortality. Other functional measures, such as grip strength [9] and Short Physical Performance Battery [10], have also been associated with a risk of cardiometabolic disease (e.g., diabetes, hypertension, and hypercholesterolemia) and mortality.

TUG test results were also found to predict the incidence of CV disease, CV mortality, and all-cause mortality. For example, TUG results were found to predict 3-year all-cause mortality in patients with chronic kidney disease [11], and to be associated with increased long-term all-cause mortality in older adults [12, 13]. Moreover, the TUG test was reported to be the best physical function test to predict all-cause mortality and CV disease in older men [14].

To our knowledge, however, previous studies were limited to specific population (e.g. male sex, chronic kidney disease) with small number of participants, and no studies to date have assessed these relationships in large general populations. Most of the studies examined the relationship between TUG test and all-cause mortality, and only few studies have assessed the relationship between TUG test results and rates of CV disease and CV mortality. Consequently, there is limited generalizability of relationship between TUG test and CV disease and mortality in general population.

This study evaluated the associations of TUG test results in subjects aged 66 years with CV disease and mortality in a large general population of subjects enrolled in the National Screening Program for Transitional Age (NSPTA) in Korea, which is a nationwide representative sample of Korean people.

## Methods

### Study design

This study was retrospective cohort study using the National Health Information Database (NHID) in Korea. The study participants were 66 years old men and women when they participated in NSPTA program in 2007–2008. The observation started from the date they received examination in the program, and ended on the date of outcome event for participants who experienced the event (i.e. cardiovascular event, cardiovascular death, all-cause death) or December 31, 2013 for participants who did not experience the event. We aimed to compare the events between normal and abnormal group in TUG results using survival analysis.

### Data sources

The Korean National Health Insurance Service (KNHIS) created the NHID, which includes data on healthcare utilization, health screening, sociodemographic variables, and mortality for over 50 million subjects in Korea [15]. The present study evaluated the senior cohort database of the NHID (2002–2013), consisting of 10% of a random sample of the Korean population aged ≥60 years, who qualified for National Health Insurance or the Medical Aid Program at the end of December 2013. Because all persons in Korea are thought to be enrolled in the National Health Insurance or Medical Aid Program, this sample is representative of older Korean adults.

**Fig. 1:**
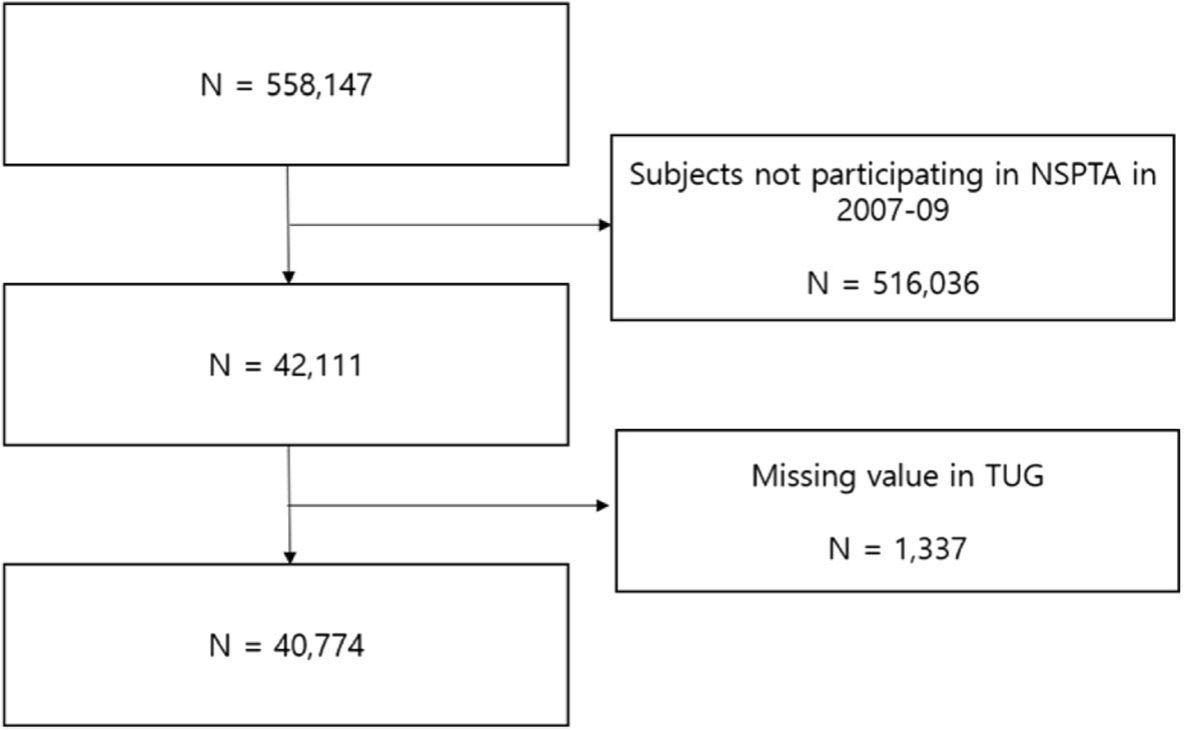
Flow of subject selection. Number of subjects in the original senior cohort database of the Korean National Health Insurance Service (2002–2013). *Abbreviation: NSPTA, National Screening Program for Transitional Ages in Korea

The NSPTA was added to the National Health Screening Program in 2007. The purposes of the NSPTA were to tailor the program according to the age and sex of each subject, and to strengthen post-examination counseling. As part of this program, only subjects aged 66 years undergo the TUG and unipedal stance tests to assess mobility. For this reason, all participants of this study were 66 years old. The details of the NSPTA have been described elsewhere [16]. In the present study, data were obtained on subjects in the database aged 66 years who participated in the NSPTA in 2007–2008 and underwent TUG tests. Of the 558,147 subjects in the database, 40,774 were further analyzed (Fig. 1).

### Timed up and go test

The TUG was performed on the day of physical examination during the NSPTA at each subject’s community hospital, as described in the NSPTA manual. Participants were required to sit on a chair, stand and walk a 3 m course at a comfortable speed, walk back to the chair and sit again, while wearing regular footwear and/or using walking aids. The time from standing up to sitting down again was measured, with a time greater than 10 s categorized as abnormal.

### Cardiovascular events, cardiovascular mortality, and all-cause mortality

Data concerning the diagnosis of a CV event, the date of the event, the cause of death, and the date of death were obtained from the senior cohort database of the KNHIS during the period 2007–2013. This analysis assumed that there was no censoring other than death or an event. Because all participants are supposed to be beneficiaries of the National Health Insurance or Medical Aid Program in Korea, drop-out other than death is virtually impossible. Furthermore, because claims for all medical events experienced by subjects should be submitted to the KNHIS by healthcare providers for reimbursement, every CV event should be included in the database. The database was reviewed for International Classification of Diseases 10th Revision (ICD-10) codes for diagnosis and cause of death. CV event or death was defined as a diagnosis of or death caused by ischemic heart diseases (I20–25) or cerebrovascular disease (I60–69). If there was no date of death in the database, the subject was considered alive at the end of 2013. The follow-up time was calculated as the time from the date of the NSPTA to the date of first diagnosis of CV disease for CV events, and to the date of death for CV and all-cause mortality.

### Potential confounders

Information on cigarette smoking was collected by a self-administered questionnaire at the time of the NSPTA, with subjects classified as current smokers, ex-smokers, or non-smokers. The questionnaire was also used to collect information on alcohol drinking and exercise. At-risk drinking was defined as drinking more than seven drinks per week or three drinks per occasion. Regular exercise was defined as vigorous activity (> 20 min/day) more than once per week. Because insurance premiums charged by the KNHIS are determined by participants’ income, insurance premium was regarded as a surrogate marker for income.

Body mass index (BMI) was calculated as weight divided by height squared (kg/m^2^). BMI was defined according to Asian-specific criteria, with normal BMI defined as between 18.5 and 23 kg/m^2^ and obesity as ≥25 kg/m^2^.

Hypertension, diabetes mellitus, dyslipidemia, and chronic kidney disease were included in analysis as cardiovascular risk factors. Subjects were regarded as having these conditions if there were records in the database that their physicians had submitted claims based on these diagnoses.

Cognition was measured using the Korean Dementia Screening Questionnaire-Cognition (KDSQ-C), which is included in the NSPTA questionnaire. The KDSQ-C is a self-administered, validated questionnaire [17], consisting of 15 items, each rated on a three-point Likert scale (0, 1, or 2, with a higher score considered worse). Cognitive impairment was defined as a composite score ≥ 6.

The NSPTA questionnaire included six items about activity of daily living (ADL), which were extracted from the Korean versions of the ADL (K-ADL) and Instrumental ADL (K-IADL) questionnaires [18]. The four items extracted from the K-ADL were: “Do you bathe by yourself without help?,” “Do you dress by yourself without help?,” “Do you eat by yourself without help if a meal is prepared?,” and “Do you go to the toilet by yourself without help?” The two items extracted from the K-IADL were “Do you prepare your meal by yourself without help?” and “Do you go outside by yourself to places within walking distance?” ADL was categorized as abnormal if the answer to one or more of these questions was “No.”

### Statistical analysis

Statistical analyses were performed using STATA software (Version 15.1; STATA. Corp, College Station, Tex). Statistical significance was defined as *P* <  0.05. Continuous variables were reported as mean ± SD and categorical variables as frequencies and percentages. To compare two groups, we used Student t-test for continuous variables and chi-square test for categorical variables. The incidence rates of CV disease, CV mortality, and all-cause mortality per 1000 person-years were calculated.

Cox proportional hazard models were used to evaluate the association of TUG results with CV events, CV mortality, and all-cause mortality. Three models were built for survival analyses, a crude model and two adjusted models. Model 1 was adjusted for sociodemographic and behavioral factors, such as sex, income, cigarette smoking, at-risk alcohol drinking, and regular exercise. Model 2 included all factors in Model 1, as well as cognitive impairment, ADL, and chronic diseases known to be CV risk factors such as obesity, hypertension, diabetes mellitus, dyslipidemia, and chronic kidney disease. Hazard ratios (HR) and 95% confidence intervals (CI) were calculated for each model.

Participants at risk with abnormal TUG results were identified by subgroup analyses in Model 2, with participants subgrouped by socio-behavioral factors, including sex, cigarette smoking, at-risk alcohol drinking, regular exercise, and chronic diseases such as obesity, hypertension, diabetes mellitus, and dyslipidemia.

## Results

### Baseline characteristics of subjects

Of the 40,774 subjects analyzed, 21,691 (53.2%) were women. Subjects were followed-up for a mean 5.7 years (maximum: 7.0 years), with 2147 (5.3%) subjects dying by the end of 2013, with only 271 (0.7%) dying of CV diseases. Abnormal TUG results (≥10 s) were observed in 15,661 (38.4%) of the 40,774 subjects. The mean BMI of the subjects was 24.2 ± 3.0 kg/m^2^, with 973 (2.4%) being underweight (BMI < 18.5 kg/m^2^) and 15,548 (38.1%) being obese (BMI ≥ 25.0 kg/m^2^).

Approximately 15% of participants were current smokers, and 30% were at-risk drinkers. Fewer than 40% reported that they exercised regularly. About 70% had been diagnosed with hypertension, whereas about 50% each had been diagnosed with diabetes and dyslipidemia. Only 3% had a history of chronic kidney disease.

Most of the participants (96.6%) had normal ADL, while three quarters had normal KDSQ-C results (Table 1).

**Table 1:** Baseline characteristics of participants

	Total	Normal	Abnormal	
	*N* = 40,774 (%)	*N* = 25,112 (%)	*N* = 15,661 (%)	*P* value^b^
Sex (women) (%)	21,690 (53.2)	12,662 (50.4)	9028 (57.7)	< 0.001
Income, lowest quintile	10,026 (24.6)	6258 (24.9)	3768 (24.1)	0.029
Timed up and go (sec)	9.4 ± 4.3	7.4 ± 1.4	12.5 ± 5.2	< 0.001
BMI (kg/m^2^)^a^	24.2 ± 3.0	24.2 ± 3.0	24.4 ± 3.1	
< 18.5	973 (2.4)	603 (2.4)	370 (2.4)	< 0.001
18.5–23	13,017 (31.9)	8140 (32.4)	4877 (31.1)	
23–25	11,236 (27.6)	7070 (28.2)	4166 (26.6)	
≥ 25	15,548 (38.1)	9299 (37.0)	6248 (39.9)	
Cigarette smoking				
Never	18.162 (70.2)	11,089 (68.5)	7072 (73.1)	< 0.001
Former	3757 (14.5)	2547 (15.7)	1210 (13.0)	
Current	3949 (15.3)	2557 (15.8)	1392 (14.0)	
At-risk alcohol drinking	12,483 (30.6)	7842 (31.2)	4641 (29.6)	< 0.001
Regular exercise	15,539 (38.1)	9694 (38.6)	5845 (37.3)	< 0.001
Hypertension	29,110 (71.4)	17,668 (70.4)	11,442 (73.1)	< 0.001
Diabetes mellitus	18,548 (45.5)	11,233 (44.7)	7315 (46.7)	< 0.001
Dyslipidemia	22,511 (55.2)	13,826 (55.1)	8685 (55.5)	0.431
Chronic kidney disease	1273 (3.1)	770 (3.1)	503 (3.2)	0.411
Normal activity of daily living	25,172 (96.6)	15,635 (96.3)	9536 (97.2)	< 0.001
Normal cognition	31,067 (77.9)	19,249 (78.5)	11,818 (76.9)	< 0.001
Cardiovascular disease	3111 (7.6)	1745 (7.0)	1366 (8.7)	< 0.001
Cardiovascular death	271 (0.7)	137 (0.6)	134 (0.9)	< 0.001
All-cause death	2146 (5.3)	1207 (4.8)	939 (6.0)	< 0.001

^a^*Abbreviation*: *BMI* body mass index

^b^ In comparison between normal and abnormal groups, Student t-test was used for continuous variables, and chi-square test was used for categorical variables

### Cardiovascular event, cardiovascular mortality, and all-cause mortality

Incidence rates of CV events in the normal and abnormal TUG groups were 7.9 and 9.0 per 1000 person-years, respectively. Their CV mortality rates were 1.0 and 1.5 per 1000 person-years, respectively, and their all-cause mortality rates were 8.5 and 10.5 per 1000 person-years, respectively.

**Fig. 2:**
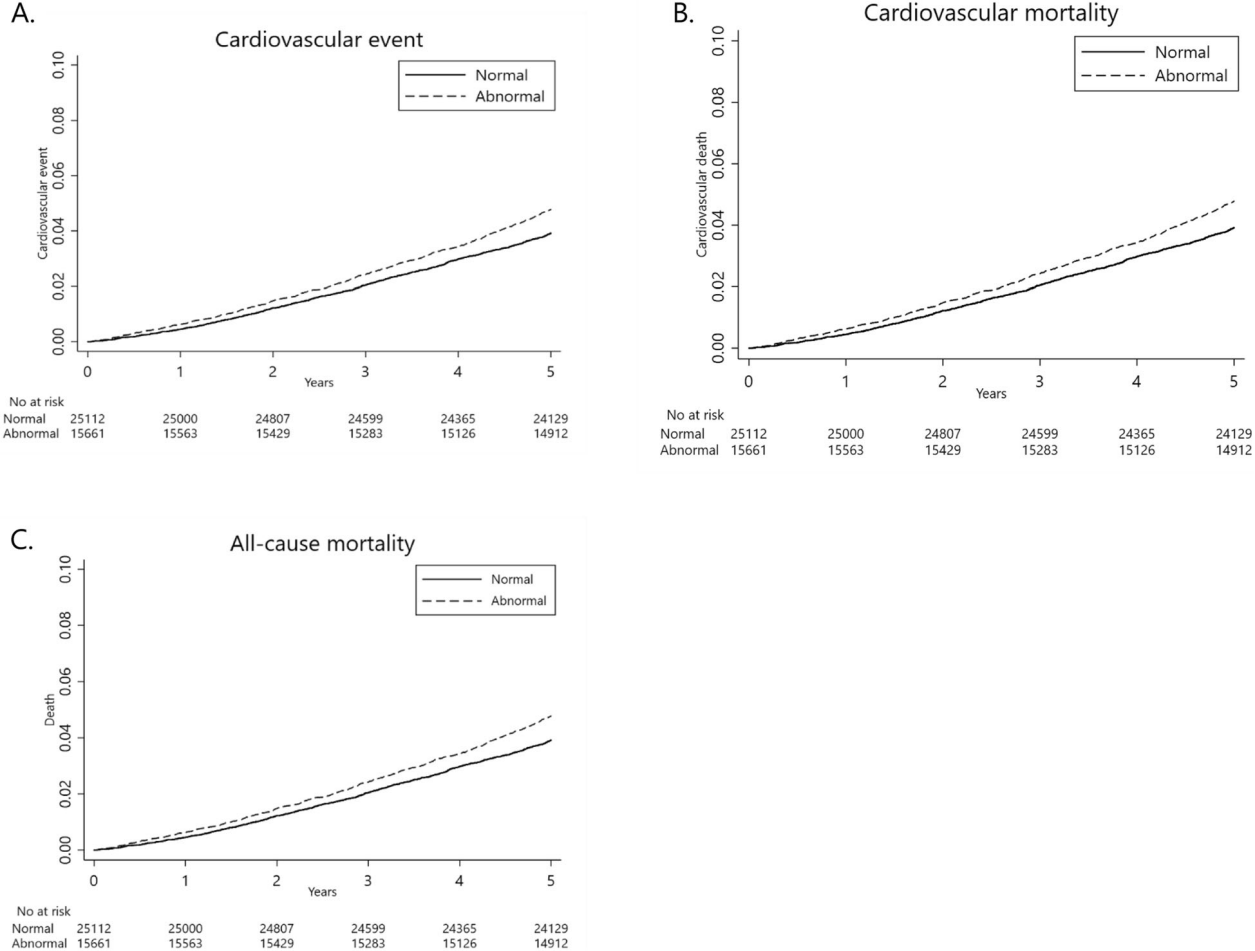
Kaplan-Meier curves of the association of timed up and go test results with **a**) cardiovascular events, **b**) cardiovascular mortality, and **c**) all-cause mortality. *Abbreviations: HR, hazard ratio; CI, confidence interval

In the crude model, CV event rates were 13% higher in subjects with abnormal than normal TUG results (HR: 1.13, 95% CI: 1.03–1.24), while CV mortality rates were 56% higher in the abnormal TUG group (HR: 1.56, 95% CI: 1.23–1.98). All-cause mortality rate was also higher in the abnormal TUG group (HR: 1.24, 95% CI: 1.14–1.35; Fig. 2, Table 2).

**Table 2:** Association of timed up and go test results with cardiovascular events, cardiovascular mortality, and all-cause mortality^a^

	TUG^c^	Number	Event	Duration (PYs)^c^	Incidence rate	Crude	Model 1^b^	Model 2^b^
						HR (95% CI)^c^	aHR (95% CI)^c^	aHR (95% CI)^c^
Cardiovascular event	Normal	25,112	1098	138,474.0	7.9	1	1	1
	Abnormal	15,661	769	85,637.3	9.0	1.13 (1.03–1.24)	1.18 (0.98–1.42)	1.09 (0.88–1.35)
Cardiovascular death	Normal	25,112	137	142,377.1	1.0	1	1	1
	Abnormal	15,661	134	89,014.7	1.5	1.56 (1.23–1.98)	1.60 (1.01–2.54)	1.59 (0.95–2.67)
All-cause death	Normal	25,112	1207	142,377.1	8.5	1	1	1
	Abnormal	15,661	939	89,014.7	10.5	1.24 (1.14–1.35)	1.25 (1.05–1.47)	1.23 (1.02–1.48)

^a^Cox proportional hazard was applied

^b^Model 1 was adjusted for sex, income, cigarette smoking, at-risk alcohol drinking, and regular exercise, whereas Model 2 was adjusted for all factors in Model 1 plus cognitive impairment, activity of daily living, body mass index, hypertension, diabetes mellitus, dyslipidemia, and chronic kidney disease

^c^*Abbreviation*: *TUG* timed up and go test, *PY* person-year, *HR* hazard ratio, *aHR* adjusted hazard ratio, *CI* confidence interval

In Model 1, the CV mortality rate was 60% higher in subjects with abnormal than normal TUG results (adjusted HR [aHR]: 1.60, 95% CI: 1.01–2.54), while the all-cause mortality rate was 25% higher in the abnormal TUG group (aHR: 1.25, 95% CI: 1.05–1.47, respectively). The difference in CV event rate between these two groups was not statistically significant.

In Model 2, however, only the all-cause mortality was significantly higher in subjects with abnormal than normal TUG results (aHR: 1.23, 95% CI: 1.02–1.48). By contrast, CV event and CV mortality rates did not differ significantly (Table 2).

### Subgroup analysis

**Fig. 3:**
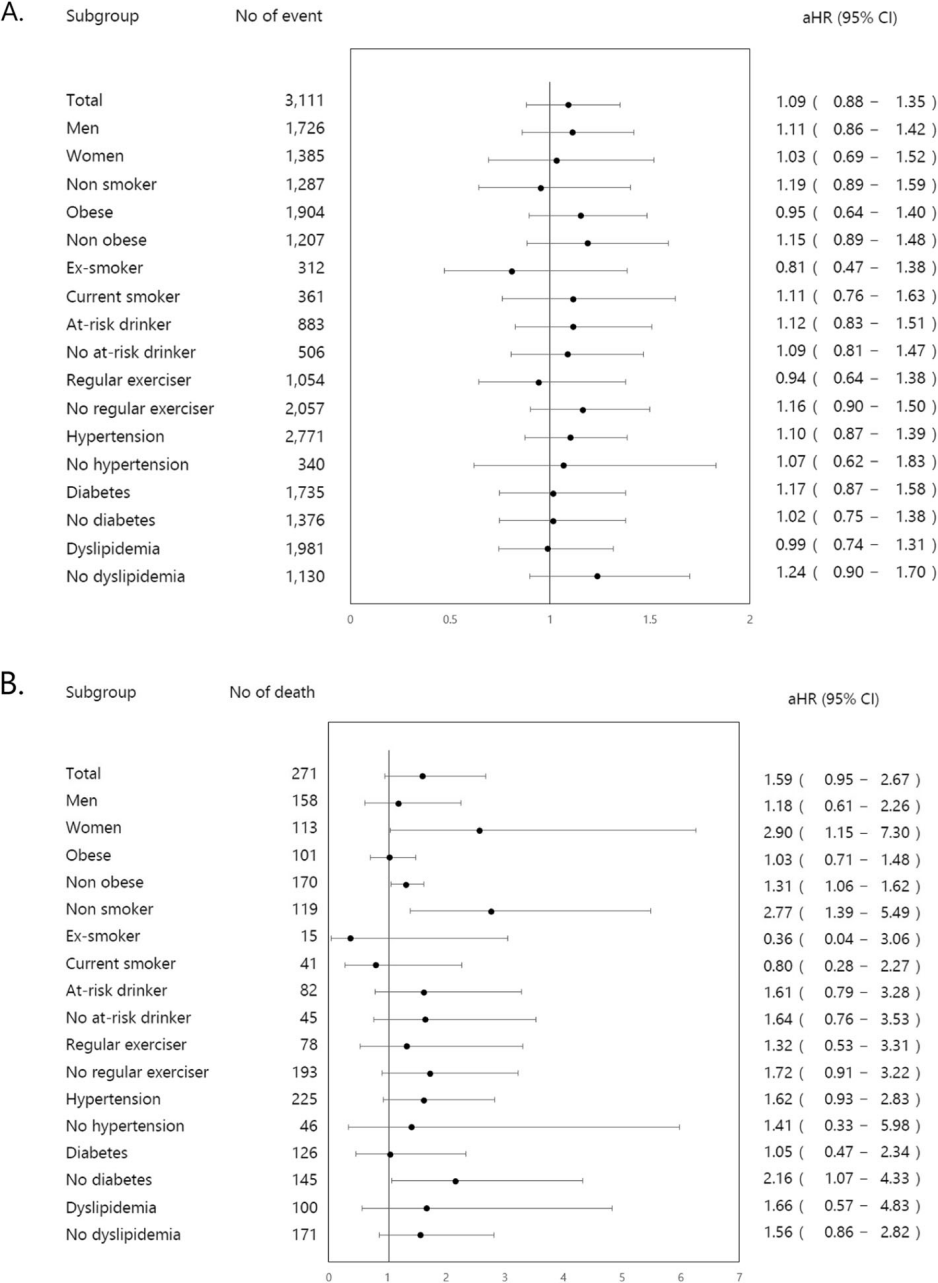
Forest plots showing subgroup analyses of the association of timed up and go test results with **a**) cardiovascular events and **b**) cardiovascular mortality. *Abbreviation: aHR, adjusted hazard ratio

Subgroup analysis failed to identify any subgroup with a significant association between TUG results and increased CV event risk. However, CV mortality rates in subjects with abnormal TUG results were higher in women (aHR: 2.56, 95% CI: 1.05–6.26), in non-obese participants (aHR: 1.31, 95% CI: 1.06–1.62), in non-smokers (aHR: 2.77, 95% CI: 1.39–5.49), and in non-diabetic participants (aHR: 2.16, 95% CI: 1.07–4.33) (Fig. 3).

## Discussion

This large longitudinal study evaluated the association between TUG test results and future incidence of CV event and mortality in a nationally representative sample of subjects in an Asian country. Using a fully adjusted model, we found that abnormal TUG results were not associated with the incidences of CV events and CV mortality. However, abnormal TUG results (≥10 s) resulted in a 2.5-fold increase in CV mortality in women, whereas no increase was observed in men. Further, participants lacking certain CV risk factors, such as current cigarette smoking, obesity, or diabetes, had a higher CV mortality rate when TUG results were abnormal.

This study is the first large general population-based longitudinal study evaluating the association between TUG results and CV disease and CV mortality. Previous studies regarding this association were limited to specific population, or examined the association only with all-cause mortality. Therefore, this study elucidated that the association between TUG results and CV mortality is generalizable, especially in women. Furthermore, this study finding implies that there is discrepancy of the association by sex and CV risk profiles.

Abnormal TUG results were associated with all-cause mortality in both sexes (data not shown). These results are similar to the results of previous studies concerning TUG and all-cause mortality. For example, TUG results were found to predict 3-year all-cause mortality in 385 chronic kidney disease patients of mean age 61 years [13]. Furthermore, the all-cause mortality rate was 79% higher in subjects with the slowest than the fastest fifth of TUG results after follow-up for up to 11.8 years [11]. In addition, a study in 300 older women found that a one second increase in TUG test results was associated with a 10% increase in all-cause mortality after follow-up for 13.5 years [12].

In contrast to a study reporting that TUG predicted the incidence of CV disease in older men, we found that an abnormal TUG result was not associated with CV incidence or mortality in men [14]. There are three possible explanations for this discrepancy. First, the population was older, and the follow-up duration was longer in the previous study, suggesting that the association between CV disease and TUG results varies with age. Second, the model in the previous study was adjusted only for BMI and smoking status. However, when we built and analyzed a model adjusted only for BMI and smoking status in men, the result was similar to that obtained with Model 2 (aHR: 1.07, 95% CI: 0.91–1.27). Third, the population in the previous study was Caucasian, whereas our population was Asian. The incidence of CV is higher in Caucasians than in Asians, suggesting that ethnic differences in CV incidence and mortality may have led to a discrepancy between the results of the two studies.

Physical performance has been shown to be associated with markers of CV disease. Additionally, sarcopenia, which is important physiology of physical frailty, is related to disability, hospitalization, and death [19]. Some CV markers, such as carotid artery intima media thickness [20] and serum concentrations of homocysteine [21] and high-density lipoprotein cholesterol [22], are associated with walking speed. Moreover, inflammatory markers, which are risk factors for CV disease [23]. These findings suggest that CV disease and physical performance in older adults have common risk factors and disease mechanisms. In addition, preclinical CV disease was shown to precede clinical frailty [24], suggesting that persons with abnormal TUG results may have had significant but silent preclinical vascular alterations.

CV mortality rates in the present study differed in men and women. After adjustment, the association of abnormal TUG results with CV mortality was significant only in women, indicating that men and women differ in risk factors for CV disease. This discrepancy may be partly explained by differences in sex hormone. Although low testosterone levels in older men is associated with falls and poorer physical performance [25], low testosterone levels in men aged < 70 years were not significantly associated with CV disease [26]. In women, menopause is a well-established CV risk factor, but mean grip strength was lower in postmenopausal women aged 53 years than in pre- or perimenopausal women [27]. These findings indicate that sex hormones in both sexes influence CV risk and physical performance in different ways.

Although CV mortality was not significantly associated with TUG results in the entire study population, CV mortality rates were higher in participants lacking certain CV risk factors, such as obesity, diabetes, and current cigarette smoking, suggesting that physiologic changes associated with TUG abnormality are not a strong risk of CV mortality compared with conventional risk factors. Thus, TUG results would not affect CV mortality rates in participants with these risk factors. However, the 2-fold higher rate of CV mortality in participants with abnormal TUG results lacking strong risk factors such as cigarette smoking and diabetes suggests that TUG results may help identify a higher risk among subjects without strong CV risk factors.

This study had several limitations. The study population included only 66-year-old men and women in Korea, preventing assessment of the association of TUG results with outcomes in different age groups. Caution should be exercised in applying the results of this study to older populations. The limitation of the database prevented adjustment for well-known risk factors of CV incidence or mortality, such as education and dietary intake. Additionally, because the database did not include the information about severity of CV diseases, we could not control for severity. Moreover, the mean follow-up duration of 5.7 years was relatively short compared with previous studies evaluating all-cause mortality, which may lead to a lack of statistical power. Therefore, there is need for longer follow-up studies that include missing confounders. Lastly, as NHID is basically a database for medical claims, there could be misclassifications of diagnoses of CV disease and cause of death. However, every claim in this dataset was audited by the Korean Health Insurance Review and Assessment before payment, making the misclassification of diagnosis improbable.

## Conclusions

Abnormal TUG results in subjects aged 66 years were associated with future CV mortality in women and in subjects without obesity, diabetes, or cigarette smoking. In patient with mobility impairment, physicians should consider CV disease risk, especially in women.

## Data Availability

The dataset generated and analyzed during the current study are available in National Health Insurance Sharing Service. But Authors have no right to share or provide the data. The information of how to request for database is available in https://nhiss.nhis.or.kr/bd/ab/bdaba021eng.do. And detail and cost of the database is described in https://nhiss.nhis.or.kr/bd/ab/bdaba022eng.do. To request the database, visit https://nhiss.nhis.or.kr/bd/ay/bdaya001iv.do. (only available in Korean). The questionnaire used in this study is not available in English. Korean version of the questionnaire is available for download in following website: (http://www.law.go.kr/admRulLsInfoP.do?chrClsCd=&admRulSeq=2200000012541#AJAX).

## Abbreviation

ADL: Activity of daily living
aHR: Adjusted hazard ratio
BMI: Body mass index
CI: Confidence interval
CV: Cardiovascular
HR: Hazard ratio
IADL: Instrumental activity of daily living
KDSQ-C: Korean Dementia Screening Questionnaire-Cognition
KNHIS: Korean National Health Insurance Service
NHID: National Health Information Database
NSPTA: National Screening Program for Transitional Ages
TUG: Timed Up and Go
